# Development of a Multiplex Immunohistochemistry Workflow to Investigate the Immune Microenvironment in Mouse Models of Inflammatory Bowel Disease and Colon Cancer

**DOI:** 10.3390/ijms222011001

**Published:** 2021-10-12

**Authors:** Lokman Pang, Matthias Ernst, Jennifer Huynh

**Affiliations:** Olivia Newton-John Cancer Research Institute, School of Cancer Medicine, La Trobe University, Heidelberg, VIC 3084, Australia; matthias.ernst@onjcri.org.au

**Keywords:** multiplex immunohistochemistry, tumour microenvironment, immune infiltration, immune cells, inflammatory bowel diseases, colon cancer

## Abstract

Multiplex immunohistochemistry (mIHC) enables simultaneous staining of multiple immune markers on a single tissue section. Mounting studies have demonstrated the versatility of mIHC in evaluating immune infiltrates in different diseases and the tumour microenvironment (TME). However, the majority of published studies are limited to the analysis of human patient samples. Performing mIHC on formalin-fixed paraffin-embedded (FFPE) mouse tissues, particularly with sensitive antigens, remain challenging. The aim of our study was to develop a robust and reproducible protocol to uncover the immune landscape in mouse FFPE tissues. Effective antibody stripping while maintaining sensitivity to antigens and tissue adhesion to the glass slide is critical in developing an mIHC panel to allow successive rounds of staining. Thus, we identified a highly efficient stripping method that preserves signal intensity and antigenicity to allow multiple rounds of staining. We subsequently optimised an mIHC workflow with antibodies specific against CD4, CD8α, FOXP3 and B220 to identify distinct T and B cell populations on mouse FFPE tissues. Lastly, the application of this mIHC panel was validated in a mouse model of inflammatory bowel cancer, two allograft mouse models of spontaneous colon adenocarcinoma and a sporadic mouse model of colon cancer. Together, these demonstrate the utility of the aforementioned protocol in establishing the quantity and spatial localisation of immune cells in different pathological tissues.

## 1. Introduction

Understanding the immune microenvironment is fundamental in investigating the pathogenesis of different diseases as well as improving the understanding of immune cell behaviour and interaction in the tumour microenvironment (TME). Although standard chromogenic immunohistochemistry (IHC) is fundamental in research and diagnostic pathology, this technique only allows the detection of a single marker. Immunophenotyping for more than one marker with standard chromogenic IHC remains impractical as this requires serial tissue sections from often precious samples, thereby resulting in reduced spatial information. Meanwhile, multiplex immunohistochemistry (mIHC) has emerged as a widely adopted technique to simultaneously evaluate multiple markers on a single tissue section through consecutive rounds of staining. This technique overcomes the hurdle of standard chromogenic IHC and enables the use of multiple unlabelled primary antibodies, regardless of the species the primary antibodies are raised in. mIHC utilises sequential rounds of primary antibodies labelling of one marker, followed by the addition of horseradish peroxidase (HRP)-conjugated secondary antibodies. This in turn catalyses the formation of covalent bonds of multiple tyramide-fluorophore complexes to the tyrosine residues proximal to the antigen of interest [1,2]. This technology allows primary and secondary antibodies to be removed from the section before the next round of staining with a different primary antibody without the risk of cross-reactivity [3,4]. Thus, mIHC significantly improves staining efficiency and preserves the spatial context across different immune cells and its underlying tissue.

In the context of cancer, mIHC provides valuable insight into the relationship between infiltrating immune cells and tumour cells in the TME. The accuracy and efficiency of mIHC in reflecting the composition of the immune microenvironment have been demonstrated in both human specimens [5,6,7,8] and animal mouse models [2,9]. However, established mIHC protocols predominately focused on human samples, and its use in preclinical animal models remain limited [2,9]. It is also important to note that the study of human biopsy samples remains limited for several reasons. Human malignancies often develop over a long period and are identified later in life, thereby limiting the study of the biological events involved in cancer initiation and progression. Given that repeated sampling from human patients is often not feasible for ethical reasons, the use of rapid and reproducible preclinical mouse models serves as a valuable tool to investigate disease pathogenesis and to validate novel therapeutic strategies. In particular, mouse models of inflammatory bowel disease (IBD) and colon cancer have been instrumental in dissecting disease mechanisms and advanced our understanding of the immune microenvironment [10,11,12]. Emerging studies have demonstrated that cell localisation can dramatically influence the clinical stage and outcome of colon cancer [13,14,15]. This highlights the need to establish a standardised technique to quantitatively analyse the immune microenvironment in mouse models of IBD and colon cancer. Nonetheless, several studies suggest that certain murine immune markers such as CD4, CD8α are difficult to detect as these epitopes are highly sensitive to many fixation methods and/or heat-mediated antigen retrieval [2,16,17]. Repeated rounds of microwave treatment (MWT), for instance, are detrimental to tissue integrity, slide adhesion and damages antigens, thereby precluding further rounds of staining [18,19].

Although zinc-based fixation has been demonstrated to be effective in preserving these antigens and reduce epitope masking, formalin remains as the most conventional tissue preservation method for histological analyses in mouse tissues [17,20,21,22,23,24]. Here, we optimised an alternative antibody stripping method to maximise preservation of tissue integrity, adhesion and antigenicity on mouse formalin-fixed paraffin-embedded (FFPE) samples. Based on this method, we developed an mIHC panel with specific markers to identify different T and B cell populations. Finally, we confirmed the robustness of our protocol by determining the immune microenvironment in preclinical mouse models of inflammatory bowel disease and colon cancer.

## 2. Results

### 2.1. Standard Chromogenic IHC and Opal Monoplex Assay Development

**Figure 1:**
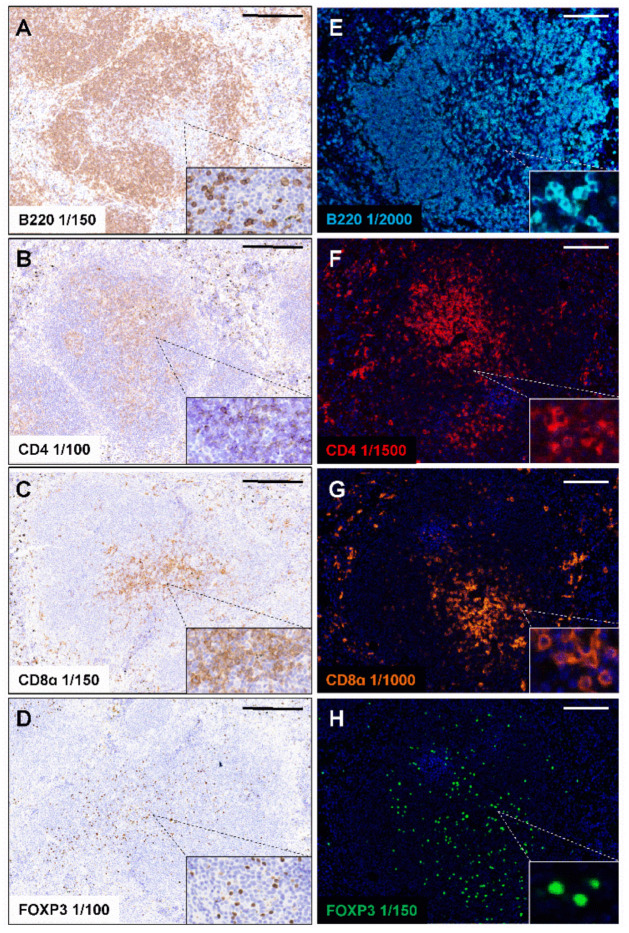
Standard chromogenic IHC and Opal monoplex staining of each immune marker on FFPE mouse spleens. Formalin-fixed paraffin-embedded (FFPE) C57BL/6 mouse spleens were stained with antibodies specific against B220 (**A**), CD4 (**B**), CD8α (**C**) and FOXP3 (**D**) using standard chromogenic immunohistochemistry (IHC) and counterstained with haematoxylin. Scale bar: 200 µm. Opal monoplex staining of B220 (**E**), CD4 (**F**), CD8α (**G**) and FOXP3 (**H**) were performed on FFPE C57BL/6 mouse spleens and counterstained with DAPI. Tissue sections were imaged at 20× magnification on Vectra 3. B220 (cyan); CD4 (red); CD8α (orange); FOXP3 (green). Scale bar: 100 µm.

We first validated four immune markers specific for different B and T cell subsets using chromogenic IHC on mouse FFPE spleen sections. These immune markers include B220 (pan-B cells; also known as cluster of differentiation 45 or CD45R), CD4 (helper T cells; Cluster of differentiation 4), CD8α (cytotoxic T cells; Cluster of differentiation 8α) and FOXP3 (regulatory T cells; Forkhead box P3), which are expressed in cells in the spleen as well as in the inflamed colon and TME during tumorigenesis [22,25]. Citrate Buffer pH 6.0 is commonly used in heat-mediated antigen retrieval and was sufficient for the staining of B220, CD4 and CD8α in FFPE mouse spleens (Figure 1A–C). However, antigen retrieval in citrate buffer was insufficient for the staining of FOXP3, resulting in minimal signal (Supplementary Figure S1). It has been suggested that antigen retrieval buffers with higher pH may improve antigen unmasking than the lower pH citrate buffer [26]. We, therefore, assessed the ability of Tris-EDTA pH 9.0 buffer to unmask the FOXP3 antigen. We found that Tris-EDTA buffer pH 9.0 was necessary to generate robust FOXP3 staining in FFPE mouse spleens (Figure 1D). Using standard chromogenic IHC, we validated the conditions of antigen retrieval required and the ability of each primary antibody to produce robust and uniform staining in mouse FFPE spleens.

Next, we performed Opal monoplex staining for each primary antibody to assist in determining the staining parameters for each individual primary antibody in the subsequent multiplex panel. In comparison to standard chromogenic IHC, the Opal tyramide signal amplification technology employed in mIHC significantly enhances sensitivity and specificity, thereby enabling more diluted use of primary antibodies [27]. This allowed the detection of CD4, CD8α and B220 on the cell membrane and nuclear expression of the transcription factor FOXP3 as expected (Figure 1E–H). These data suggest that the monoplex IHC can be transferred to mIHC to facilitate the identification of different B and T cell populations in mouse tissues.

### 2.2. Comparison of Different Antibody Stripping Methods

**Figure 2:**
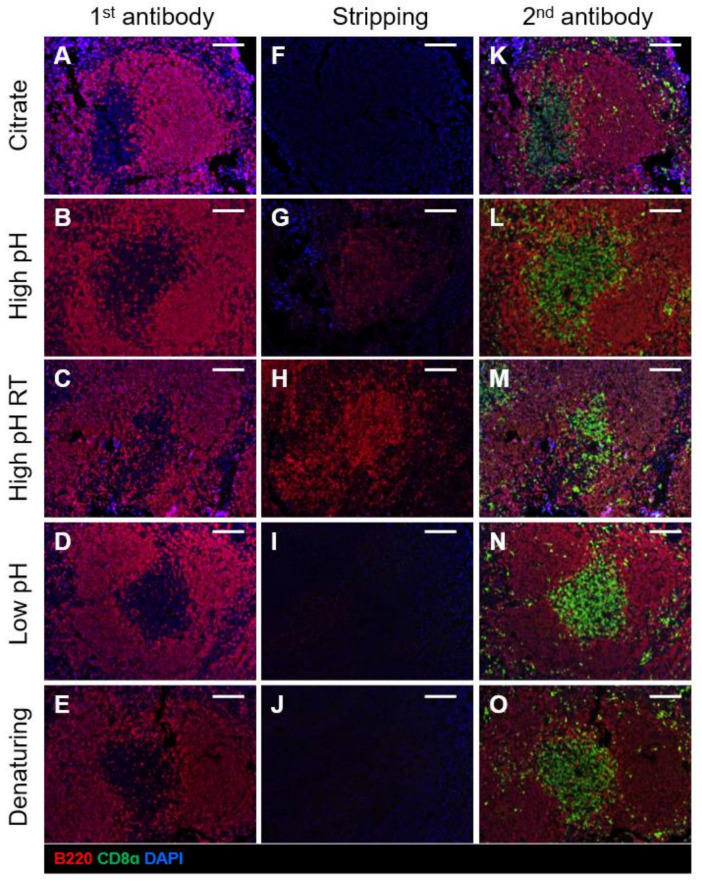
Assessment of antibody stripping methods. FFPE mouse spleen sections were stained with B220 (red) as the first antibody (**A**–**E**), followed by stripping methods listed below. Stripping efficiency was evaluated after microwave treatment in citrate buffer (**F**), incubation in high pH glycine-based buffer at 50 °C (**G**) or room temperature (**H**), low pH glycine-based buffer at 50 °C (**I**) and glycine-SDS denaturing buffer at 50 °C (**J**). To test whether antigenicity or signal intensity of the previous marker are affected by the stripping method, sections were stained with a second antibody CD8ɑ (green) (**K**–**O**). Tissue sections were imaged at 20X magnification on Vectra 3. RT: room temperature. Scale bar: 100 µm.

As repeated rounds of MWT can profoundly affect tissue integrity, adhesion to the slide and antigenicity, we sought to identify an alternative antibody stripping method that was efficient in the removal of the bound antibody complex. As outlined in Table 1, several antibody stripping methods have been proposed in the literature [16,28,29]. These methods include incubating slides in glycine-based buffers with high pH (pH 10.0), low pH (pH 2.2), and denaturing conditions using sodium dodecyl sulphate (SDS). A previous study demonstrated that the B220 antigen was more resistant to repeated rounds of MWT, whereas the ability to stain for CD8α was more labile after heating [2]. Thus, we tested first whether these stripping methods could successfully remove strong antigens such as B220. Mouse FFPE spleen sections were stained with a primary antibody against the pan-B cell marker B220 (Figure 2A–E), followed by treatment with each antibody stripping method. MWT in citrate buffer was effective in stripping antibodies (Figure 2F) as predicted. Some studies have reported effective stripping by incubating slides in a high pH glycine-based stripping buffer (pH 10.0) at 50 °C or room temperature [16,28]. However, we found that incubation in a high pH buffer at 50 °C resulted in residual signal (Figure 2G) and failed to remove the antibody complex at room temperature in our laboratory settings (Figure 2H). By contrast, slide incubation in a low pH glycine-based stripping buffer (pH 2.2) and a glycine-SDS denaturing buffer (pH 2.0) for 30 min at 50 °C both showed no remnant signal after incubation with Opal fluorophore and were highly efficient in the removal of the bound antibody complex (Figure 2I,J).

We then assessed whether these antibody stripping methods influenced the ability to detect subsequent markers or the signal intensity for the previous marker. We, therefore, stained slides with a primary antibody against the sensitive antigen, CD8α. We found that antigenicity was not affected across all methods (Figure 2K–O), although these slides have only undergone one round of MWT antigen retrieval and antibody stripping at this stage. However, we found that MWT in citrate buffer and incubation in the glycine SDS denaturing buffer significantly reduced signal intensity of the marker tested prior to antibody stripping (Figure 2K,O, Supplementary Figure S2). Meanwhile, incubation in the low pH glycine-based buffer showed minimal signal loss (Figure 2N). Based on these results, we concluded that antibody stripping using the low pH glycine-based stripping buffer was the most efficient in removing the antibody complex while preserving signal intensity.

### 2.3. Validation of Multiplex Staining Protocol on Mouse Spleen and Different Disease Models

**Figure 3:**
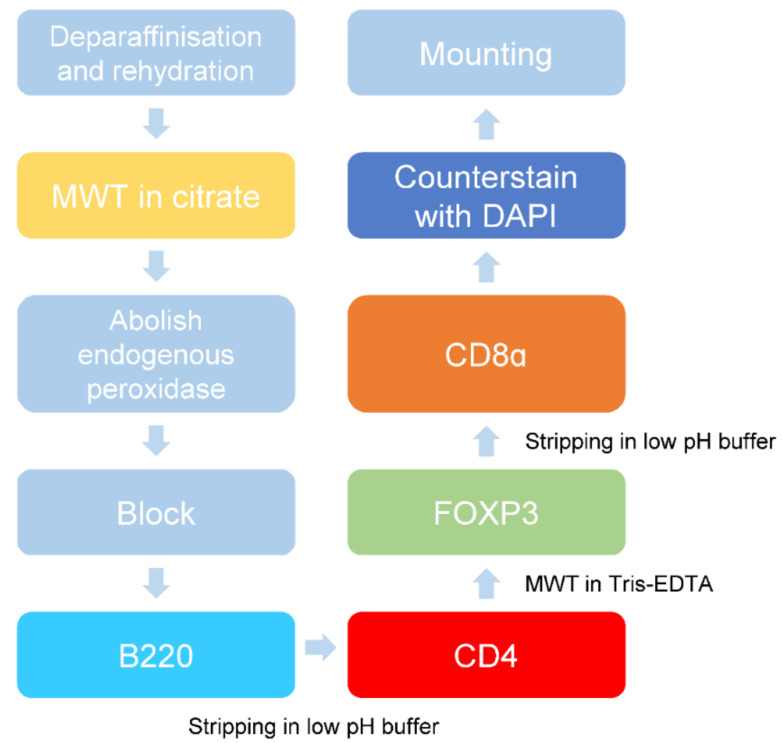
Schematic of the optimised multiplex staining workflow.

**Figure 4:**
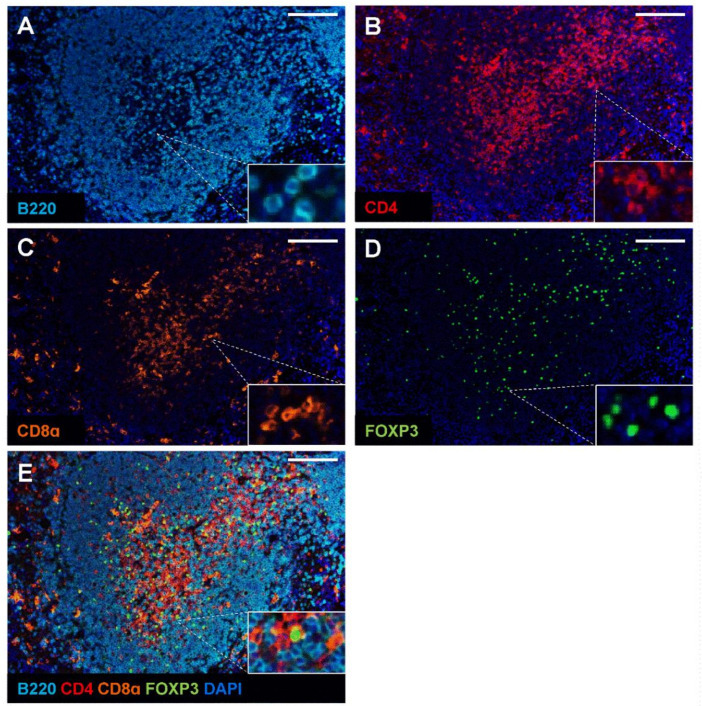
Validation of multiplex immunohistochemistry on FFPE mouse spleens. The optimised multiplex staining protocol was performed on formalin-fixed paraffin-embedded (FFPE) C57Bl/6 mouse spleen sections. Slides were stained with B220, CD4, FOXP3 and CD8α antibodies in that order with low pH glycine-based stripping buffer or Tris-EDTA antigen retrieval in between. Representative images of each channel (**A**–**D**) and the merged image (**E**) are shown. Tissue sections were imaged at 20X magnification on Vectra 3. B220 (cyan); CD4 (red); CD8α (orange); FOXP3 (green). Scale bar: 100 µm.

Next, we combined the Opal monoplex parameters and the low pH glycine-based antibody stripping method to generate a multiplex staining protocol. Opal fluorophores were assigned to each immune marker depending on its expression levels. The less abundant markers (i.e., CD4 and CD8α) were assigned the brightest fluorophores Opal 620 and Opal 570, respectively, whereas the dimmest Opal 690 fluorophore was assigned to B220 due to its increased abundance in the spleen. As spectral overlap may occur for co-localising antibodies within the same cells [21], we assigned Opal 520 to FOXP3 to prevent potential bleeding of signal in regulatory T cells that simultaneously express CD4 and FOXP3. Likewise, we relegated the Opal fluorophores such as 520 and 570 to later rounds of staining due to their tendencies for signal attenuation [21]. Mouse spleen FFPE sections were used as a positive control to determine the effectiveness of the mIHC staining panel. While the sequence of staining can significantly affect the ability to stain for the next marker [6,30], we determined the optimal staining order in mouse FFPE spleen tissue to be B220, CD4, FOXP3, followed by CD8α. Initial antigen retrieval via MWT in citrate buffer was required to unmask antigens. Subsequent staining and the antibody complex were stripped in the low pH glycine-based buffer. An additional round of MWT in Tris-EDTA was incorporated as a stripping buffer while improving access to the intracellular antigen FOXP3. The stepwise protocol and antibody concentrations used are summarised in Table 2 and illustrated schematically in Figure 3. The multiplex images obtained from this staining sequence showed specific staining with limited background for each immune marker (Figure 4). Importantly, our optimised mIHC panel is able to discriminate and immunophenotype subsets of B and T cells in mouse spleen controls.

The optimised multiplex staining protocol involves the standard dewax and rehydration procedures, followed by microwave treatment (MWT) in citrate buffer pH 6.0. Slides are then incubated with PeroxAbolish to reduce the activity of endogenous peroxidase and Opal diluent/blocking buffer to prevent non-specific binding. Slides were first stained for B220, and the bound antibody complex is removed by stripping in low pH glycine-based buffer. This is followed by staining with CD4, MWT in Tris-EDTA pH 9.0 and subsequent FOXP3 staining. After another round of antibody stripping, slides were stained for CD8α, counterstained with DAPI and mounted for imaging.

### 2.4. Application of mIHC Panel in Mouse Models of IBD and Colon Tumour Models

**Figure 5:**
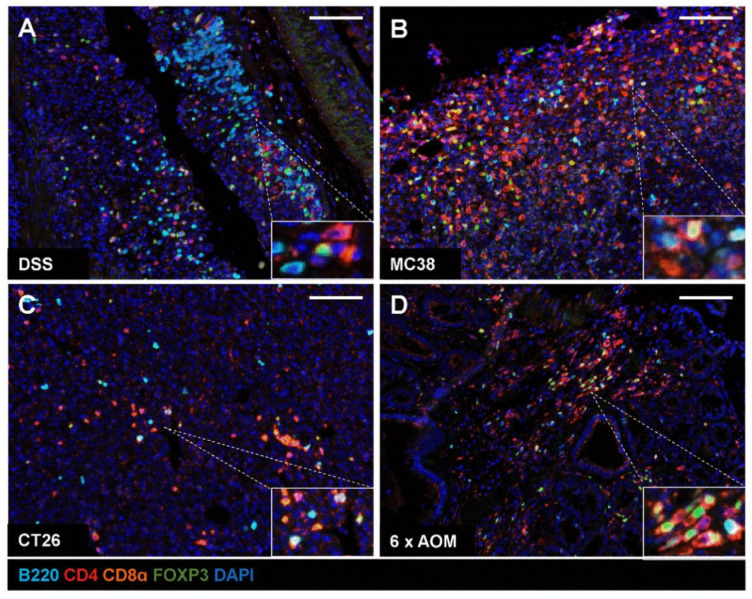
Application of the optimised multiplex staining panel in murine disease and cancer models. Multiplex staining was performed on the colon of mice treated with 3.5% dextran sulphate sodium (DSS) (**A**). Staining was also performed on mouse models injected with murine colon adenocarcinoma cells (MC38 or CT26) (**B**,**C**) or after repeated administration of the colon carcinogen azoxymethane (AOM) (**D**). Tissue sections were imaged at 20X magnification on Vectra 3. B220 (cyan); CD4 (red); CD8α (orange); FOXP3 (green). Scale bar: 100 µm.

Finally, we evaluated whether our mIHC workflow can be applied to characterise immune infiltrates in gastrointestinal models of inflammatory bowel disease and colon cancer. We employed a mouse model of inflammatory bowel disease through the administration of the chemical irritant DSS in drinking water (Figure 5A). In addition, we established allograft tumours by subcutaneously injecting the murine colon adenocarcinoma cell lines MC38 and CT26 in C57BL/6 or BALB/c recipient mice, respectively (Figure 5B,C). We further tested the application of the mIHC immune panel in a sporadic colon cancer model after mice were repeatedly exposed to the colon carcinogen AOM (Figure 5D). Tissues were harvested then stained for B220, CD4, CD8α and FOXP3 using our mIHC protocol. mIHC was efficient in distinguishing between B220^+^ B cells, CD4^+^ FOXP3^-^ helper T cells, CD8α^+^ cytotoxic T cells and CD4^+^ FOXP3^+^ regulatory T cells. Taken together, our data demonstrate the reliability of this mIHC panel to uncover immune cell infiltration in murine models of colitis and colon cancer.

## 3. Discussion

mIHC takes advantage of multiple cycles of staining and antibody stripping to allow detection of multiple markers on a single tissue section, thereby overcoming the limitation of standard chromogenic IHC. Despite the effectiveness and reliability of mIHC in simultaneously detecting several markers, most of the published mIHC protocols have been optimised for human FFPE specimens [5,31,32]. Since preclinical mouse models are indispensable in translational research, we sought to optimise a protocol and mIHC workflow for mouse FFPE tissue to characterise the immune microenvironment in intestinal disease and cancer. In the present study, we provided an optimised mIHC protocol for mouse FFPE tissue that produced robust and reproducible staining to uncover immune infiltrates in animal mouse models of IBD and colon cancer.

Performing mIHC staining for CD4 and CD8α or other sensitive epitopes on mouse FFPE tissues remains challenging and consequently is less commonly adopted in current research. A number of studies have demonstrated that certain mouse immune antigens are fixation-sensitive and are not easily detected in mouse FFPE tissues [17,21,22]. Although this limitation was historically overcome by the use of frozen tissue, the freezing process often distorts tissue architecture and results in artefacts that limit morphological studies [33]. The use of formalin-free zinc-salt fixation has been proposed and is effective in retaining the detection of sensitive epitopes [17,21]. However, most archival samples are usually fixed in formalin, which also remains the most commonly used fixation method for mouse tissues. Although flow cytometry analysis represents a valuable tool for profiling the immune landscape, this technique requires tissue digestion into a single cell suspension and limits contextual information. Steinert et al. further demonstrated that standard lymphocyte isolation for flow cytometry analysis often underestimates the number of tissue-resident memory CD8 T cells [34], highlighting the need for an improved approach to quantitatively analyse different immune cell subsets.

An efficient stripping method is essential in the mIHC process as tyramide-based Opal fluorophores are extremely sensitive to residual antibodies left from previous stripping steps. MWT in citrate or Tris-EDTA buffer is traditionally used for both antigen retrieval as well as stripping of primary and secondary antibodies after each staining cycle. However, repeated rounds of MWT often result in partial or total detachment of tissue from the glass slide, particularly with tissues derived from the skin, lung, kidney and brain [18,29]. Zhang et al. and Viratham et al. demonstrated that tyramide fluorophores were also prone to signal attenuation after multiple rounds of heating [35,36]. In our study, we identified that slide incubation in low pH glycine-based buffer (pH 2.2) at 50 °C with agitation for 30 min was the most efficient in removing the primary and secondary antibody complex and preserving signal intensity. The use of this buffer follows stripping principles commonly employed in Western blotting, whereby the acidic condition alters the structure of bound antibodies, such that their binding sites are altered and/or inactivated [37,38]. Moreover, Pivetta et al. and Willemsen et al. both demonstrated that sensitive immune markers such as CD3, CD4 and CD8α cannot sustain more than two or three rounds of MWT and result in diminished or non-specific signal [2,18]. Our study shows that antibody stripping in the low pH-glycine-based buffer does not affect antigenicity, whereby robust CD8α signal could be detected after one round of antibody stripping with no reduction in signal intensity for the previous marker B220. In comparison to conventional MWT to remove bound antibodies, stripping using the aforementioned method at mild heat preserves tissue integrity and adhesiveness.

Determining the staining sequence for each marker is fundamental in developing a robust and reproducible mIHC panel. We validated our mIHC protocol to establish a basic immune profile in the spleen of naïve mice, as well as in a model of DSS-induced colitis, two tumour allograft models and a mutagen-induced endogenous colon cancer model. To our knowledge, our study is the first to report an mIHC panel to simultaneously identify distinct populations of CD4^+^FOXP3^+^ regulatory T cells, CD4^+^ helper T cells, CD8α^+^ cytotoxic T cells and B220^+^ B cells on mouse FFPE tissues. Data generated from this panel will provide valuable insight in the quantity and spatial distribution of immune cells within the TME. It is important to note that the mIHC workflow described in the current study relied on manual processing of sections, which may introduce variability across numerous samples and human error. An automated slide stainer can be utilised to increase reproducibility and throughput [8,39,40]. We postulate that the antibody titrations as well as staining conditions can be transferred from a manual procedure to an automated platform. Furthermore, these devices are equipped with instruments to perform heat-mediated antigen retrieval. The antibody stripping step can, therefore, be achieved by substituting the standard citrate stripping buffer with the low pH-glycine stripping buffer and adjusting incubation times and temperature. However, appropriate assay development and optimisation are essential to ensure a robust and reproducible automated mIHC protocol.

While our study has predominately focused on FFPE mouse colon tissue, this protocol can easily be implemented to study the contextual significance of immune cells in other murine pathological tissues. Given that mIHC staining by Opal chemistry can accommodate up to six markers, it is possible to add or substitute other markers of interest to the existing panel, which can be tailored to accommodate different research needs. Stringent optimisation and validation will allow wide adaptation of mIHC to elucidate the immune microenvironment and its contribution to different diseases in preclinical mouse models.

## 4. Materials and Methods

### 4.1. Animals

We used 6–8 week old mice raised on a C57BL/6 or BALB/c background, which were housed in individually ventilated cages (IVC) with a 12:12 h light:dark cycle under specific pathogen-free conditions. All animal experiments were conducted in accordance with the Animal Ethics Committee of Austin Health (approval number: A2016/05325, A2016/05327, A2016/05418).

### 4.2. DSS-Induced Colitis

We induced acute experimental colitis through the administration of a chemical irritant in mice to mimic human inflammatory bowel disease as previously described [25]. Briefly, mice were administered 3.5% dextran sulphate sodium (DSS) (MW = 36–50 kDa; MP Biochemicals, Santa Ana, CA, USA) in drinking water *ad libitum* until the experimental endpoint, as determined by a 20% loss of initial bodyweight.

### 4.3. Tumour Cell Lines

We established subcutaneous syngeneic allografts through injection of the murine colon adenocarcinoma cell lines, 1 × 10^6^ MC38 cells (C57BL/6), or 5 × 10^5^ CT26 cells (BALB/c) in 100 µL PBS. For induction of sporadic colorectal cancer in mice, we administered the alkylating agent azoxymethane (AOM; 10 mg/kg, Sigma, MI, USA) once a week over a course of 6 consecutive weeks [41].

### 4.4. Tissue Fixation

Endogenous colons and allograft tumours were fixed for 24 h in 10% neutral buffered formalin then transferred to 80% ethanol. Tissues were processed for paraffin embedding and sectioned at 5 µm for subsequent staining.

### 4.5. Chromogenic Immunohistochemistry and Opal Monoplex Assay Development

For standard chromogenic IHC, slides were deparaffinised (2 × 10 min), in 100% ethanol (2 × 5 min), 70% ethanol (1 × 5 min) and ddH_2_O (1 × 5 min). Antigens were retrieved by heating in a microwave pressure cooker with 0.1% citrate buffer, pH 6.0 or Tris-EDTA pH 9.0 (10 mM Tris Base, 1 mM EDTA) for 15 min. Slides were washed thoroughly in TBST and treated with 3% hydrogen peroxide for 20 min to quench endogenous peroxidase activity. Slides were then blocked in 5% *(v/v)* goat serum for 1 h at room temperature then incubated with primary antibodies against B220, CD4, CD8α and FOXP3 (Table 3) diluted in 5% (*v/v*) goat serum overnight at 4 °C. Slides were then incubated with a biotinylated rabbit anti-rat IgG secondary antibody (Vector Laboratories, Burlingame, CA, USA) as per manufacturer’s instructions. The Diaminobenzidine (DAB) substrate Chromogen System (Dako, Brüsseler Str, Berlin, Germany) was used to develop sections and counterstain was achieved with haematoxylin. Slides were imaged with an Aperio Slide Scanner (Leica Biosystems, Melbourne, Australia).

For Opal monoplex development, slides underwent the same process for de-paraffinisation, rehydration and antigen retrieval as described above. Before the addition of primary antibodies, slides were incubated in PeroxAbolish (Biocare Medical, Pacheco, CA, USA) for 30 min to abolish the activity of endogenous peroxidase and blocked in Opal antibody diluent/blocking solution (Perkin Elmer, Waltham, MA, USA) for 10 min at RT. Slides were incubated with anti-rat secondary HRP-conjugated antibody (Vector Laboratories, Burlingame, CA, USA) followed by incubation with Opal fluorophores for 10 min at RT. Lastly, slides were counterstained with spectral DAPI for 5 min and mounted in ProLong Diamond Antifade Mountant (Thermofisher, Waltham, MA, USA). Working concentration for each antibody was determined based on uniform staining intensity and the correct staining pattern.

### 4.6. Antibody Stripping

Several antibody stripping methods have been reported in the literature [3,16,28,29]. We compared previously described antibody stripping methods [3,16,28,29], including microwave heating, extreme pH (i.e., pH 10.0, pH 2.2) and denaturing buffers (Table 1). Slides were incubated with a primary antibody against B220, followed by the addition of an anti-rat secondary HRP antibody (Vector Laboratories Burlingame, CA, USA). For microwave heating, slides were placed in 10 mM citrate buffer pH 6.0 and heated at 100% till boiling point was reached, followed by 10 min at 10% power. For the remaining methods, slides were placed in jars containing the different preheated buffers (high pH, low pH, denaturing buffer) and incubated for 30 min at RT or 50 °C with agitation. Slides were then incubated with Opal 690 for visualisation to test the efficiency of the stripping method. Additionally, we tested if the stripping method interfered with antigenicity for subsequent staining or signal intensity from the previous marker. Slides were stained with B220 and bound antibodies are removed by the stripping method described above. After a thorough wash in TBST, slides were blocked in Opal antibody diluent/blocking solution (Perkin Elmer, Waltham, MA, USA) and proceeded to staining with primary antibody against CD8α. Slides were incubated with an anti-rat secondary HRP antibody (Vector Laboratories Burlingame, CA, USA) and Opal 570 for visualisation.

### 4.7. mIHC Staining

FFPE tissue sections were de-paraffinised in xylene (2 × 10 min), rehydrated in 100% ethanol (2 × 5 min), 70% ethanol (1 × 5 min) and ddH_2_O (1 × 5 min). Antigen retrieval was performed by placing slides in 10 mM citrate buffer pH 6.0 or Tris-EDTA pH 9.0 (10 mM Tris Base, 1 mM EDTA) according to the requirement of the primary antibodies and heated in a microwave for 1 min on 100% power then 10 min on 10% power. Slides were washed in TBST (3 × 2 min) and incubated in PeroxAbolish (Biocare Medical, Pacheco, CA, USA) for 30 min to block endogenous peroxidase, followed by a thorough wash in TBST (3 × 2 min). Non-specific binding was then blocked with Opal antibody diluent/blocking solution (Akoya Biosciences, Marlborough, MA, USA) for 10 min at RT. Primary antibodies against B220, CD4, CD8α or FOXP3 (Table 3) were diluted in Opal antibody diluent/blocking solution incubated at RT for 1 h or at 4 °C overnight. Slides were washed in TBST (3 × 2 min) and incubated with anti-rat secondary HRP-conjugated antibody (Vector Laboratories Burlingame, CA, USA) for 10 min at RT. For fluorescence detection, an Opal 7-colour manual IHC kit (Akoya Biosciences, Marlborough, MA, USA containing the Opal 520, Opal 570, Opal 620 and Opal 690 fluorophores was used. Slides were incubated with the appropriate Opal fluorophore for 10 min in the dark, followed by TBST wash (3 × 2 min). Multiplex staining was achieved by removing the antibody complex after incubation in low pH-glycine buffer (pH 2.2) at 50 °C for 30 min with agitation. After a thorough wash in TBST, slides were blocked in Opal antibody diluent/blocking solution and staining cycles were repeated. Once all the targets were stained, slides were counterstained with spectral DAPI for 5 min at RT, washed in TBST (3 × 2 min) and coverslipped in ProLong Diamond Antifade Mountant (Thermofisher, Waltham, MA, USA). A summary of the staining sequence, the concentration of primary antibodies and Opal fluorophores are found in Table 2.

### 4.8. Image Acquisition and Analysis

Stained slides were imaged using the Vectra 3 automated quantitative pathology imaging system 3.0.5 (Akoya Biosciences, Marlborough, MA, USA). Acquired images were opened in inform 2.4.1 (PerkinElmer, Waltham, MA, USA) and spectrally unmixed using the appropriate spectral library.

## 5. Conclusions

In summary, our study has evaluated an alternative low pH antibody stripping approach to overcome the traditional challenges of heat-mediated antigen retrieval. We detailed the workflow of an mIHC panel on murine FFPE tissues to allow identification of immune cell infiltrates in a quantitative manner while preserving its spatial context. This protocol can be easily implemented in other laboratory settings to elucidate the significance of the immune microenvironment in different pathological tissues.

## Figures and Tables

**Table 1:** List of antibody stripping methods.

Method	Buffer	Incubation Time
Microwave	10 mM citrate, pH 6.0	1 min on 100% power (till boiling point is reached), followed by 7.5 min on 10% power
High pH	100 mM glycine NaOH, pH 10.0	50 °C 30 min or RT 15 min
Low pH	50 mM glycine HCl, pH 2.2	50 °C 30 min
Denaturing	25 mM glycine HCl, 10% SDS, pH 2.0	50 °C 30 min

**Table 2:** Multiplex staining panel for the identification of T and B cell subsets.

Staining Order	Antigen Retrieval	Primary Antibody	Secondary Antibody	Opal Fluorophore	Stripping
1	Citrate pH 6.0	B220 (1/2000) 1 h RT	Anti-rat secondary HRP 10 min	690 10 min	Low pH glycine buffer 30 min, 50 °C
2	-	CD4 (1/1000) Overnight 4 °C	Anti-rat secondary HRP 10 min	620 10 min	-
3	Tris-EDTA pH 9.0	Foxp3 (1/150) 1 h RT	Anti-rat secondary HRP 10 min	520 10 min	Low pH glycine buffer 30 min, 50 °C
4	-	CD8α (1/1000) Overnight 4 °C	Anti-rat secondary HRP 10 min	570 10 min	-

**Table 3:** List of primary antibodies.

Antibody	Supplier	Catalogue No.	Species	IHC Dilution	mIHC Dilution
B220	BD Pharmingen	550286	Rat	1/150	1/2000
CD4	eBioscience	14-9766-82	Rat	1/100	1/1500
CD8α	eBioscience	14-0808	Rat	1/150	1/1000
FOXP3	eBioscience	14-5773-80	Rat	1/100	1/150

## Data Availability

The data that support the findings of this study are available from the corresponding authors upon reasonable request.

## Supplementary Materials

The following are available online at https://www.mdpi.com/article/10.3390/ijms222011001/s1.
