# Supplementary material for: Development of a Multiplex Immunohistochemistry Workflow to Investigate the Immune Microenvironment in Mouse Models of Inflammatory Bowel Disease and Colon Cancer
Source: Int J Mol Sci. 2021 Oct 12;22(20):11001. doi: 10.3390/ijms222011001 (PMC8539370; doi:10.3390/ijms222011001)
Supplement: Supplementary file 1 [file ijms-22-11001-s001.zip › ijms-1400296-supplementary.pdf]

Supplementary Materials

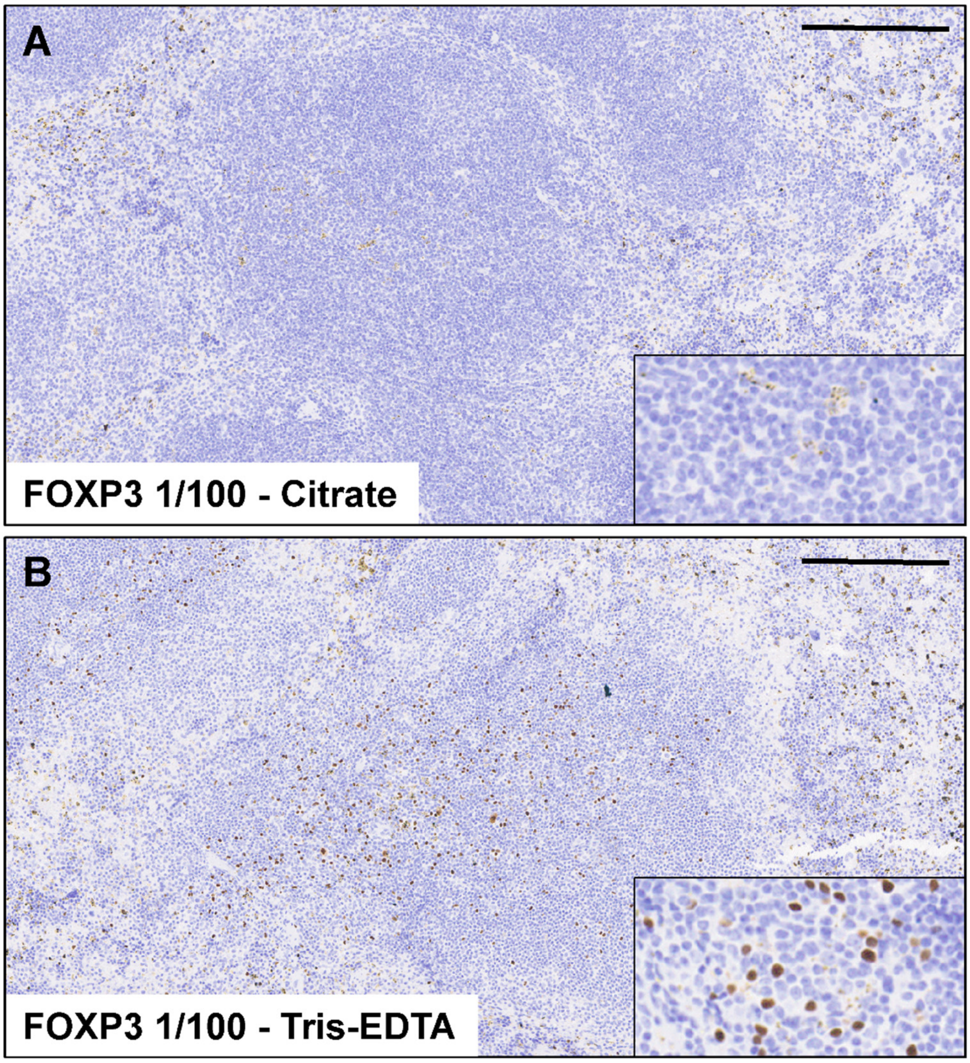

Supplementary Figure S1. FOXP3 staining requires antigen retrieval in Tris-EDTA buffer. Formalin-fixed paraffin embedded (FFPE) C57BL/6 mouse spleens were stained with an antibody specific against FOXP3 after antigen retrieval in either citrate buffer pH 6.0 (A) or Tris-EDTA buffer pH 9.0 (B), followed by counterstain with haematoxylin. Scale bar: 200μm.

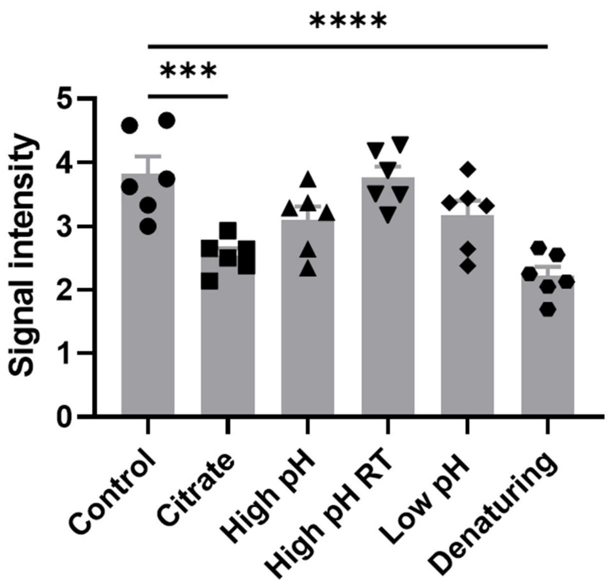

Supplementary Figure S2. Comparison of signal intensity after each stripping method. Signal intensities for B220 staining were quantified using inform v2.5.0 and compared in sections pre and post stripping. Data are analysed using one-way ANOVA with Dunnett's multiple comparisons test and represented as mean ± SEM. *** $p < 0.001$; **** $p < 0.0001$.
